# Surgical options of the breast and clinical outcomes of breast cancer patients after neoadjuvant chemotherapy: A single-center retrospective study

**DOI:** 10.3389/fonc.2022.984587

**Published:** 2022-10-27

**Authors:** Yuting Sang, Xujie Zhou, Weiru Chi, Jiajian Chen, Benlong Yang, Shuang Hao, Xiaoyan Huang, Guangyu Liu, Zhimin Shao, Jiong Wu

**Affiliations:** ^1^ Department of Breast Surgery, Shanghai Cancer Center, Fudan University, Shanghai, China; ^2^ Department of Oncology, Shanghai Medical College, Fudan University, Shanghai, China; ^3^ Human Phenome Institute, Fudan University, Shanghai, China

**Keywords:** breast cancer, neoadjuvant chemotherapy, surgery, mastectomy, breast conservation, oncoplastic surgery

## Abstract

**Background:**

Neoadjuvant chemotherapy (NAC) has evolved significantly and has been widely accepted for downstaging disease in early-stage and locally advanced breast cancer patients. Since the optimal surgical intervention for patients receiving NAC remains controversial, we aim to investigate the survival outcome of patients treated with different surgical management.

**Methods:**

A retrospective, nested case-control study was conducted in patients with invasive breast cancer that underwent NAC at Fudan University Shanghai Cancer Center from January 2010 to June 2019. Based on surgical intervention, patients were divided into mastectomy and breast conservation groups. Patients were matched on age at diagnosis, menopausal status, the year of the surgery, post neoadjuvant therapy pathological tumor (ypT) stage, post neoadjuvant therapy pathological node (ypN) stage, molecular subtypes, and axillary surgery by propensity score matching.

**Results:**

A total of 2080 patients were enrolled in this study. Among them, 1819 (87.5%) patients were categorized as mastectomy group, and 261 (12.5%) patients were classed as breast conservation group. Over 9-years of research, the proportion of breast conservation steadily increased in patients after NAC. Data showed that younger (P<0.001) and pre-menopausal (P<0.001) patients with normal BMI (P=0.022) were more likely to receive breast conservation. Patients at advanced ypT stage (P<0.001), ypN stage (P<0.001), and clinical TNM stage (P<0.001) were more often to undergo mastectomy, while breast conservation rate was significantly higher in patients with triple-negative tumors (P=0.023). Compared with the mastectomy group, significant benefits in overall survival were observed in patients who received breast conservation (Hazard ratio 0.41, [95% confidence interval: 0.18-0.97]; p=0.049) in the matched cohort. There was no statistical difference between groups related to disease-free survival and locoregional recurrence.

**Conclusions:**

Tumor biology can significantly impact the surgical decision in patients administrated with NAC. Breast conservation was a safe alternative for mastectomy in the NAC setting without compromising survival outcomes and locoregional control.

## Introduction

Neoadjuvant chemotherapy (NAC) was initially applicated to locally advanced breast cancer in the 1970s (1). Since NAC could effectively downstage tumor burden in breast and axilla and allow an early evaluation of systematic therapy, it is increasingly used with extended indications for early-stage breast cancer (2, 3). With the evolvement modern of drugs and targeted therapy, dramatic improvement in rates of pathological complete response (pCR) was observed in patients receiving NAC, which was positively correlated with improved overall survival and disease-free survival (4, 5). Based on the National Cancer Database (NCDB) data, the overall proportion of NAC uses significantly increased from 15.7 to 26.0% from 2010 to 2015 for all subtypes, especially in triple-negative and HER2-positive tumors (6).

Although previous studies indicated that patients received NAC showed no difference in survival outcomes and locoregional recurrence rate compared with those who received adjuvant chemotherapy, it may contribute to a de-escalation of local treatment, which was repeatedly raised in the present therapeutic pattern (7). Thereby, NAC brought a shift of surgical management towards a less extensive paradigm in both axilla and breast surgery (8). According to the data from two early randomized studies, the National Surgical Adjuvant Breast and Bowel Project (NSABP) B-27 and the European Organization Research and Treatment of Cancer (EORTC) Trial 10902, the rate of breast-conserving surgery (BCS) was suggested increase up to 59.8% and 23% following NAC in patients initially recommended with mastectomy (9, 10). A meta-analysis enrolled in 14 prospective researches also suggested that NAC was correlated with a definite reduction in mastectomy rate by 16.6% (11).

Since the main purpose of breast-conserving surgery was to only remove tumors without resecting normal breast tissue, patients treated with BCS were proven to have favorable cosmetic outcomes, breast satisfaction, and quality of life. Moreover, BCS was identified with equivalent survival outcomes compared with mastectomy, even as the preferred treatment in early-stage breast cancer (12–14). Whereas, considering the difficulties in the clinical and radiologic assessment of residual tumors after NAC, accurate tumor size and localization were hard to accomplish. Based on previous studies, the NSABP B-18, B-27, and the EORTC 10902, BCS after NAC was correlated with a higher risk of locoregional disease (9, 10). However, a large retrospective study involving 6134 patients with operable or locally advanced breast cancer receiving NAC suggested that breast conservation could be a safe alternation of mastectomy in terms of local recurrence-free survival, even in patients with multifocal tumors (15). Therefore, whether those advantages of BCS could be maintained remains controversial in the NAC setting.

Additionally, regarding that oncoplastic surgical techniques were increasingly accepted in patients with breast cancer, the application of oncoplastic approaches in patients treated with NAC also gained a lot of attention. For patients who were not feasible for BCS, nipple-sparing mastectomy (NSM) or skin-sparing mastectomy (SSM) with immediate breast reconstruction (IBR) may be an alternative to conventional mastectomy, which identified with the improvement of aesthetic results and quality of life (16). From the perspective of breast conservation, oncoplastic breast-conserving surgery (OBCS) emerged as a surrogate option for conventional breast-conserving surgery with larger excised volume, lower margin involvement, and comparable cosmetic outcomes (17). Considerable amounts of studies focused on the feasibility of oncoplastic surgical interventions in patients without primary chemotherapy, whereas little was conducted to evaluate their oncological safety in the NAC settings.

Therefore, to address the optimal surgical intervention after NAC and influence factors for patient selection, we conducted this retrospective study to give an overview of the present therapeutic pattern of breast surgery, and compare the survival outcomes among different surgical groups in this matched case-control study.

## Materials and methods

### Inclusion and exclusion criteria of patients

A total of 2552 female patients diagnosed with primary invasive breast cancer receiving neoadjuvant chemotherapy between January 2010 and June 2019 at Fudan University Shanghai Cancer Center (FUSCC) were enrolled. After excluding patients with distant metastasis (n=329), bilateral tumor (n=24), missing assessment of tumor immunohistology (n=76), and without surgical intervention following neoadjuvant chemotherapy (n=43), 2080 patients met the criteria and served as the study population. Based on surgical intervention performed after NAC, patients were divided into mastectomy (n=1819) and breast conservation groups (n=261). To be specific, the mastectomy group involved patients who received conventional mastectomy (CM) or mastectomy plus immediate breast reconstruction (M+IBR), while the breast conservation group evolved patients who underwent conventional breast-conserving surgery (CBCS) and oncoplastic breast-conserving surgery (OBCS). Surgical interventions were performed by highly qualified breast surgeons who had a long period of professional training in oncoplastic surgery. All the oncoplastic breast-conserving surgeries involved in this study were accomplished by volume displacement approach, which consisted of extent lumpectomy and using the remaining breast tissue to remedy the deformation resulting from tumor resection. In addition, M+IBR surgeries were accomplished by nipple-sparing mastectomy (NSM) or skin-sparing mastectomy (SSM) combined with immediate breast reconstruction (IBR). NSM was conducted under the condition that patients without nipple involvement clinically pre-operation, and the retro-areolar frozen-section specimens were confirmed to be tumor-free intra-operation.

### Data extraction

Demographic, clinical, pathological, and treatment information were retrospectively collected by medical record review. All the patients were followed up through outpatient interviews or telephone calls. The survival status of patients was extracted from the Department of Clinical Statistics of FUSCC based on the medical records or telephone follow-up records. Overall survival (OS) was measured from the date of starting NAC to the date of the last follow-up records or death from any causes. Disease-free survival (DFS) was calculated from the date of starting NAC to the date of first locoregional recurrence, metastatic relapse, or death. Local regional recurrence (LRR) was defined from the date of starting NAC to the date of first locoregional recurrence (relapse at ipsilateral breast or chest wall, in ipsilateral axillary, supra- and/or infra-clavicular, and/or internal mammary lymph nodes).

### Clinicopathological characteristics

All the patients receiving NAC were pathological proven primary invasive breast cancer by core-needle biopsy, supplemented by mammography, breast and axillary ultrasound, and magnetic resonance imaging (MRI). The selection criteria for NAC treatment was patients diagnosed with stage II-III breast cancer, while some patients subsequently identified with synchronous distant metastasis were excluded in the current study. The NAC regimens were administrated based on National Comprehensive Cancer Network Guidelines (NCCN) by the same team of treating oncologists, which generally included, taxanes or anthracycline combined with cyclophosphamide, taxanes combined with anthracycline and cyclophosphamide, and taxanes combined with platinum. Patients diagnosed with HER2-positive received HER2-targeted therapy in addition to chemotherapy. Since the radiological methods for residual tumor assessment were not unified earlier time in the study, response assessment was accomplished by pathological diagnosis after surgery. The radiotherapy following surgery depended on the surgical choice and pathological diagnosis after surgery. Endocrine therapy was offered to patients with estrogen receptor-positive or progesterone receptor-positive tumors. All of the pathological variables were determined according to the same guidelines at any time point. The staging of the tumor and axilla were categorized by the eighth edition of the American Joint Committee on Cancer (AJCC) Tumor Node Metastasis (TNM) stage criterion. Clinical T (cT) and N (cN) stages were evaluated by physical examination or imaging techniques (ultrasound, mammography, and magnetic resonance imaging) supplemented by pathological measurements. The clinical prognostic (cTNM) stage was applied to patients staging before NAC treatment. Hormone receptor (HR) status, including estrogen receptor (ER) and progesterone receptor (PR), were identified using immunohistochemistry (IHC). Human epidermal growth factor receptor-2 (HER2) positivity was defined as IHC 3+ or amplification by fluorescence *in situ* hybridization (FISH). Accordingly, tumors were divided into four molecular subtypes: (I) the luminal A-like subtype (HR-positive, HER2-negative, and Ki-67 ≤ 20%); (II) the luminal B-like subtype (HR-positive and HER2-positive; or HR-positive, HER2-negative, and Ki-67>20%); (III) the HER2-positive subtype (HER2-positive and HR-negative); (IV) the triple-negative subtype (negative ER, PR, and HER2). Pathologic complete response (pCR) is defined as the presence of *in situ* cancer after treatment in the absence of residual invasive disease in the breast, while in absence of tumor cells (invasive or *in situ*) in the axilla. Negative margins for breast-conserving surgery were defined as no ink on the tumor.

### Statistical analysis

The chi-square test and Fisher’s exact test were used to compare the demographic characteristics and clinicopathological data among groups. To minimize the bias caused by differences in clinical features and variations in treatments throughout time, we employed 1:1 propensity score matching (PSM) without replacement to balance the significant variables between groups. Patients were chosen and matched based on the age at diagnosis, menopausal status, the year of the surgery (Patients were divided into five groups based on the year of surgery, from 2010 to 2011, from 2012 to 2013, from 2014 to 2015, from 2016 to 2017, and from 2018 to 2019), post-neoadjuvant therapy pathological tumor (ypT) stage, post-neoadjuvant therapy pathological node (ypN) stage, molecular subtypes, and axillary surgery. Overall survival (OS), disease-free survival (DFS), and locoregional recurrence (LRR) were analyzed by Kaplan-Meier analysis and compared with the log-rank test. 5-year OS, 5-year DFS, and 5-year LRR were measured according to the description of Greenwood’s variance estimated by Anderson et al. (18). The hazard ratio (HR) and 95% confidence interval (95%CI) were estimated using the Cox proportional hazards regression model. A p-value <0.05 was considered statistically significant. All of the statistical analysis was carried out with IBM SPSS, version 27.0.

### Ethical approval

The study was conducted in accordance with the Declaration of Helsinki (as revised in 2013). This research has been approved by the Medical Ethics Committee of Fudan University Shanghai Cancer Center (1905202-7). Informed consent has been waived by the ethics committee as this retrospective research poses no risk to patients.

## Results

### Trends of surgical intervention in patients who underwent NAC

**Figure 1:**
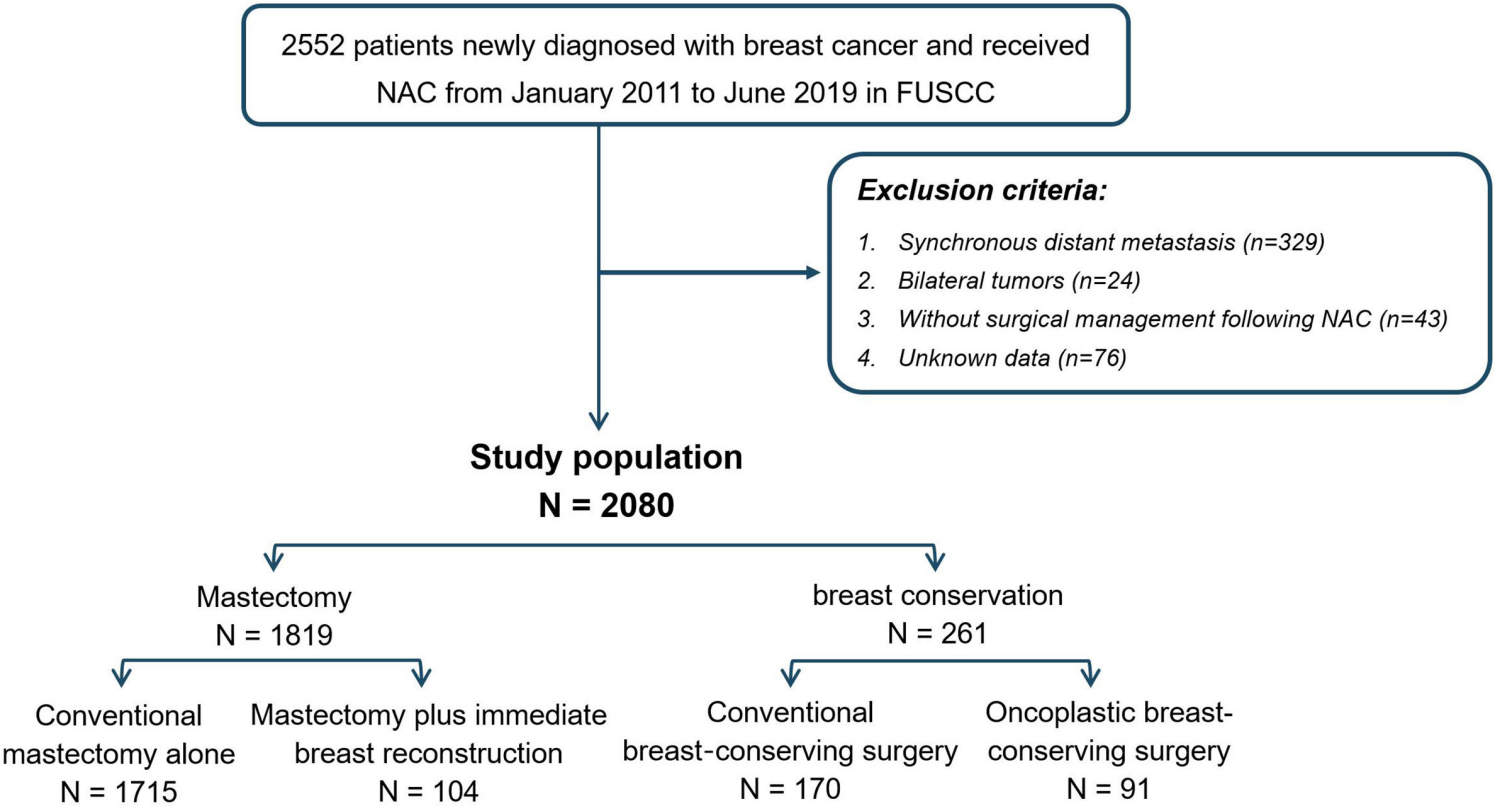
Flowchart of patient selection.

**Figure 2:**
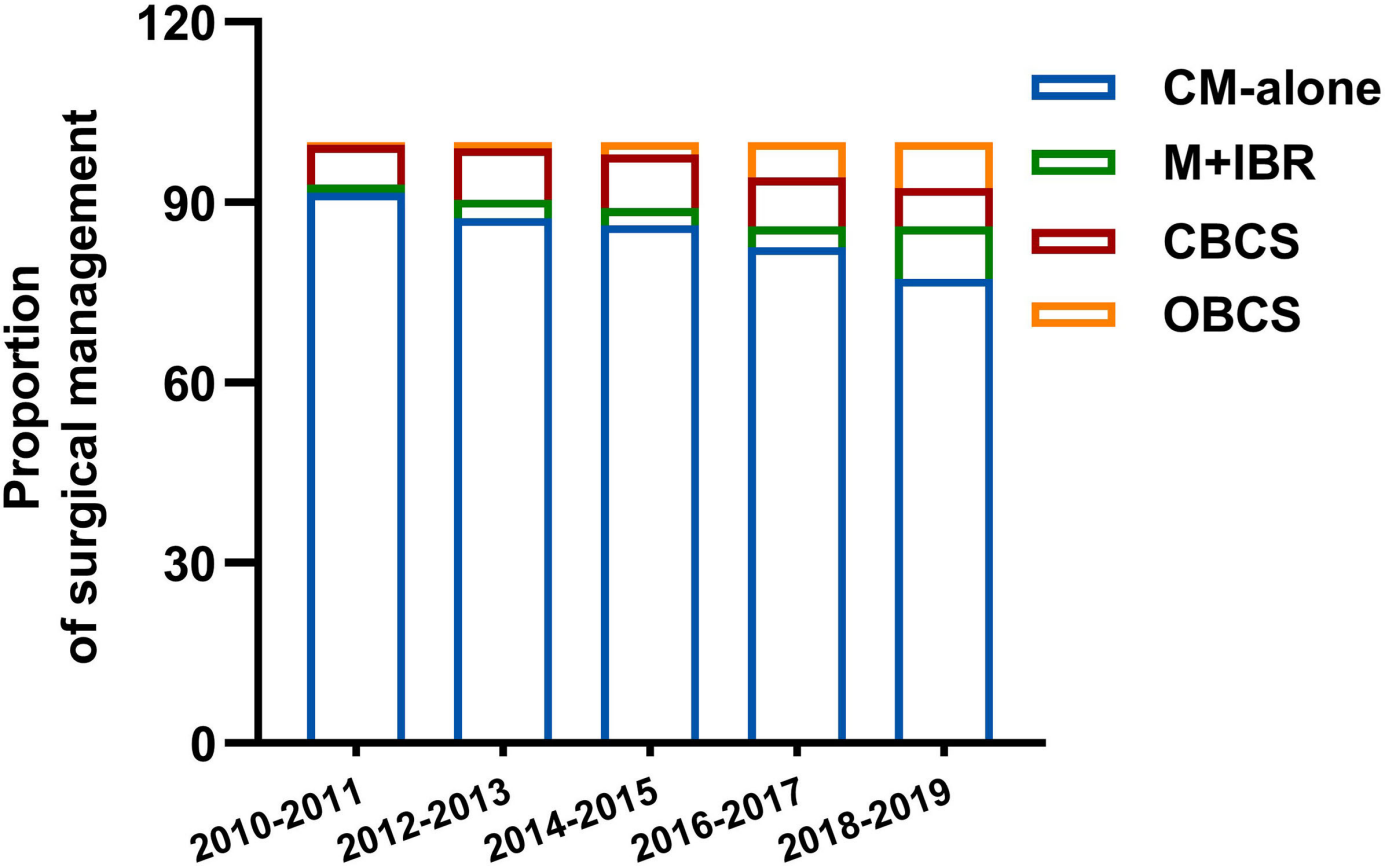
Distribution of surgical management in different period. CM-alone, conventional mastectomy alone; M+IBR, mastectomy plus immediate breast reconstruction; CBCS, conventional breast-conserving surgery; OBCS, oncoplastic breast-conserving surgery.

Among 2552 patients diagnosed with primary invasive breast cancer and received neoadjuvant chemotherapy (NAC), 2080 patients were involved in this study according to the inclusion and exclusion criteria. Among them, 1819 (87.5%) patients were categorized as mastectomy group, and 261 (12.5%) patients were classed as breast conservation group. In the mastectomy group, patients who underwent conventional mastectomy (CM) and M+IBR accounted for 82.5% (1715/2080) and 5% (104/2080) patients respectively. As for breast conservation, 170 (8.2%) and 91 (4.4%) patients were treated with CBCS and OBCS after NAC (**Figure 1**). Data showed that the proportion of breast conservation gradually increased in patients treated with NAC over the 9-years of research. Notably, the research cohort also suggested a growing prevalence of M+IBR from 1.26% to 8.70%, while fewer patients opted for CM-alone as surgical intervention after NAC. Moreover, the percentage of patients undergoing OBCS increased from 0.42% to 7.60% throughout the 9 years (**Figure 2**).

### Demographics and clinicopathological characteristics of patients.

Data indicated that demographics and clinicopathological characteristics are various among the surgical groups (**Table 1**). Patients in the mastectomy group were older (p<0.001), pre-menopausal (p<0.001), and had normal BMI (p=0.022). In addition, a larger proportion of advanced clinical T stage (p<0.001), clinical TNM stage (p<0.001), ypT stage (p<0.001), ypN stage (p<0.001), as well as non-pCR rate (p<0.001) were also observed in patients receiving mastectomy. While the rate of breast conservation was higher in patients with triple-negative tumors (p=0.023). Over 90% (1672/1819) of patients in the mastectomy group received axillary lymph node dissection (ALND), while 62.1% (162/261) of patients were conducted with sentinel lymph node biopsy (SLNB) in the breast conservation group. No significant differences were identified in the cN stage, ER status, PR status, and HER2 status among the surgical groups.

**Table 1:** Characteristics of patients in mastectomy and breast conservation groups.

Characteristics	Cases	Mastectomy (%)	Breast conservation (%)	*P*value
**No. of patients**	**2080**	**1819 (87.5)**	**261 (12.5)**	
**Age at diagnosis (years)**				< 0.001
< 55		1324 (72.8)	219 (83.9)	
≥55		495 (27.2)	42 (16.1)	
**BMI (kg/m^2^)**				0.022
Underweight (< 18.5)		53 (2.9)	4 (1.5)	
Normal weight (18.5-25)		1207 (66.4)	195 (74.7)	
Overweight (> 25)		559 (30.7)	62 (23.8)	
**Menopausal status**				< 0.001
Pre-menopausal		932 (51.2)	175 (67.0)	
Post-menopausal		887 (48.8)	86 (33.0)	
**cT stage**				< 0.001
T0/T1		165 (9.1)	49 (18.8)	
T2		1081 (59.4)	176 (67.4)	
T3/T4		573 (31.5)	36 (13.8)	
**cN stage**				0.168
N0		466 (25.6)	81 (31.0)	
N1/N2		1021 (56.1)	138 (52.9)	
N3		332 (18.3)	42 (16.1)	
**cTNM stage**				< 0.001
II		952 (52.3)	166 (63.6)	
III		867 (47.7)	95 (36.4)	
**pCR**				< 0.001
Yes		465 (25.6)	107 (41.0)	
No		1354 (74.4)	154 (59.0)	
**ypT stage**				< 0.001
T0/Tis		621 (34.1)	122 (46.7)	
T1		622 (34.2)	115 (44.1)	
T2		511 (28.1)	24 (9.2)	
T3		65 (3.6)	0 (0.0)	
**ypN stage**				< 0.001
N0		890 (48.9)	178 (68.2)	
N1		450 (24.7)	57 (21.8)	
N2		315 (17.3)	19 (7.3)	
N3		163 (9.0)	5 (1.9)	
Not Available^a^		1 (0.1)	2 (0.8)	
**ER status**				0.086
Positive		1064 (58.5)	138 (52.9)	
Negative		755 (41.5)	123 (47.1)	
**PR status**				0.306
Positive		891 (49.0)	119 (45.6)	
Negative		928 (51.0)	142 (54.4)	
**HER2 status**				0.404
Positive		731 (40.2)	97 (37.2)	
Negative		1088 (59.8)	164 (62.8)	
**Molecular subtypes**				0.023
Luminal A-like		254 (14.0)	32 (12.3)	
Luminal B-like		863 (47.4)	113 (43.3)	
HER2-positive		374 (20.6)	48 (18.4)	
Triple-negative		328 (18.0)	68 (26.1)	
**Axillary surgery**				< 0.001
Sentinel lymph node biopsy		146 (8.0)	97 (37.2)	
Axillary lymph node dissection		1672 (91.9)	162 (62.1)	
Without surgical intervention		1 (0.1)	2 (0.7)	

a Patients did not receive axillary surgery after NAC.

BMI, Body Mass Index; cT stage, clinical tumor stage; cN stage, clinical node stage; ypT stage, post neoadjuvant therapy pathological tumor stage; ypN stage, post neoadjuvant therapy pathological node stage; pCR, pathological complete response; ER, estrogen receptor; PR, progesterone receptor; HER2, human epidermal growth factor receptor 2.

The bolded values represent a total number or percentage of the study cohorts or groups.

Among the patients in the mastectomy group, data indicated that a larger proportion of younger (p<0.001) and premenopausal (p<0.001) patients with low or normal BMI (p<0.001) received M+IBR. The rate of M+IBR was significantly higher in patients with ER-positive (p=0.013), and PR-positive (p=0.042) tumors. There was no statistically significant difference in tumor and nodal stage at presentation or after NAC, HER2 status, and molecular subtypes of the tumor, along with the type of axillary surgery between patients who received CM-alone and M+IBR. In addition, the baseline of patients and tumor biologic characteristics did not significantly differ across patients underwent CBCS and OBCS (**Table 2**).

**Table 2:** Comparison of characteristics of patients receiving different surgical interventions in mastectomy and breast conservation groups.

Characteristics	Mastectomy group		*P* value	Breast conservation group		*P* value
	CM-alone	M+IBR		CBCS	OBCS	
**No. of patients**	**1715**	**104**		**170**	**91**	
**Age at diagnosis (years)**			<0.001			0.900
<55	1227 (71.5)	97 (93.3)		143 (84.1)	76 (83.5)	
≥55	488 (28.5)	7 (6.7)		27 (15.9)	15 (16.5)	
**BMI (kg/m^2^)**			<0.001			0.730
Underweight (<18.5)	44 (2.6)	9 (8.7)		2 (1.2)	2 (2.2)	
Normal weight (18.5-25)	1131 (65.9)	76 (73.1)		129 (75.9)	66 (72.5)	
Overweight (>25)	540 (31.5)	19 (18.3)		39 (22.9)	23 (25.3)	
**Menopausal status**			<0.001			0.168
Pre-menopausal	844 (49.2)	88 (84.6)		109 (64.1)	66 (72.5)	
Post-menopausal	871 (50.8)	16 (15.4)		61 (35.9)	25 (27.5)	
**cT stage**			0.114			0.053
T0/T1	152 (8.9)	13 (12.5)		42 (24.7)	15 (16.5)	
T2	1029 (60.0)	52 (50.0)		112 (65.9)	59 (64.8)	
T3/T4	534 (31.1)	39 (37.5)		16 (9.4)	17 (18.7)	
**cN stage**			0.072			0.972
N0	441 (25.7)	25 (24.0)		51 (30.0)	28 (30.8)	
N1/N2	953 (55.6)	68 (65.4)		91 (53.5)	49 (53.8)	
N3	321 (18.7)	11 (10.6)		28 (16.5)	14 (15.4)	
**cTNM stage**			0.083			0.347
II	889 (51.8)	63 (60.6)		112 (65.9)	58 (63.7)	
III	826 (49.2)	41 (39.4)		58 (34.1)	33 (36.3)	
**pCR**			0.399			0.855
Yes	443 (25.8)	23 (22.1)		69 (40.6)	38 (41.8)	
No	1272 (74.2)	81 (77.9)		101 (59.4)	53 (58.2)	
**ypT stage**			0.777			0.717
T0/Tis	589 (34.3)	32 (30.8)		79 (46.5)	43 (47.3)	
T1	582 (33.9)	40 (38.5)		77 (45.3)	38 (41.8)	
T2	482 (28.1)	29 (27.9)		14 (8.2)	10 (11.0)	
T3	62 (3.6)	3 (2.9)		0 (0.0)	0 (0.0)	
**ypN stage**			0.862			0.177
N0	836 (48.7)	54 (51.9)		119 (70.0)	59 (64.8)	
N1	429 (25.0)	21 (20.2)		32 (18.8)	25 (27.5)	
N2	296 (17.3)	19 (18.3)		15 (8.8)	4 (4.4)	
N3	153 (8.9)	10 (9.6)		2 (1.2)	3 (3.3)	
Not Available^a^	1 (0.1)	0 (0.0)		2 (1.2)	0 (0.0)	
**ER status**			0.013			0.624
Positive	991 (57.8)	73 (70.2)		88 (51.8)	50 (54.9)	
Negative	724 (42.2)	31 (29.8)		82 (48.2)	41 (45.1)	
**PR status**			0.042			0.898
Positive	830 (48.4)	61 (58.7)		78 (45.9)	41 (45.1)	
Negative	885 (51.6)	43 (41.3)		92 (54.1)	50 (54.9)	
**HER2 status**			0.323			0.393
Positive	694 (40.5)	37 (35.6)		60 (35.3)	37 (40.7)	
Negative	1021 (59.5)	67 (64.4)		110 (64.7)	54 (59.3)	
**Molecular subtypes**			0.101			0.784
Luminal A-like	241 (14.1)	13 (12.5)		22 (12.9)	10 (11.0)	
Luminal B-like	802 (46.8)	61 (58.7)		71 (41.8)	42 (46.2)	
HER2-positive	360 (21.0)	14 (13.5)		30 (17.6)	18 (19.8)	
Triple-negative	312 (18.2)	16 (15.4)		47 (27.6)	21 (23.1)	
**Axillary surgery**			0.329			0.583
SLNB	143 (8.3)	13 (12.5)		66 (38.8)	36 (39.6)	
ALND	1571 (91.6)	91 (87.5)		102 (60.0)	55 (60.4)	
Without surgery	1 (0.1)	0 (0.0)		2 (1.2)	0 (0.0)	

a Patients did not receive axillary surgery after NAC.

CM-alone, conventional mastectomy alone; M+IBR, mastectomy plus immediate breast reconstruction; CBCS, conventional breast-conserving surgery; OBCS, oncoplastic breast-conserving surgery; BMI, Body mass index; cT stage, clinical tumor stage; cN stage, clinical node stage; ypT stage, post neoadjuvant therapy pathological tumor stage; ypN stage, post neoadjuvant therapy pathological node stage; pCR, pathological complete response; ER, estrogen receptor; PR, progesterone receptor; HER2, human epidermal growth factor receptor 2; SLNB, sentinel lymph node biopsy; ALND, axillary lymph node dissection.

The bolded values represent a total number or percentage of the study cohorts or groups.

### Survival analysis between patients receiving mastectomy and breast conservation

The median follow-up time for patients in the mastectomy group was 55.0 months, and 55.2 months for the patients who underwent breast conservation. During the period of follow-up, the overall survival was 88.1% and 93.9% for patients who underwent mastectomy and breast conservation. In the mastectomy group, 81.9% (n=1490) of patients live without recurrence, with rates of recurrence were 3.2% (n=58) for locoregional recurrence, 1.0% (n=18) for local recurrence. In the breast conservation group, 88.1% (n=230) of patients live without recurrence, with rates of recurrence were 5.0% (n=13) for locoregional recurrence, 2.0% (n=5) for local recurrence. A total of 133 (6.4%) patients were lost to follow-up.

**Figure 3:**
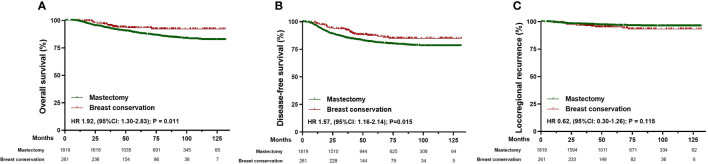
Survival analysis in the total cohort of patients receiving mastectomy (n=1819) and breast conservation (n=261). **(A)** Kaplan-Meier curves for overall survival analysis in patients of different surgical groups. **(B)** Kaplan-Meier curves for disease-free survival analysis in patients of different surgical groups. **(C)** Kaplan-Meier curves for locoregional recurrence analysis in patients of different surgical groups. HR, Hazard ratio; 95% CI, 95% confidence interval.

The overall survival (HR 1.92, [95%CI: 1.30-2.83]; p=0.011) and disease-free survival (HR 1.57, [95%CI: 1.16-2.14]; p=0.015) were higher in patients underwent breast conservation, whereas no statistically significant difference was observed in locoregional recurrence (HR 0.62, [95%CI: 0.30-1.26); p=0.115) (**Figure 3**). However, characteristics of demographics and tumor biology were considerably variable in patients who underwent different surgical management, which may lead to selection bias in the types of surgery and act as confounding factors in the comparisons of oncologic outcomes. To reduce the impact of confounding variables in comparison to survival outcomes, we developed matched cohort of patients based on age at diagnosis, menopausal status, ypT stage, ypN stage, molecular subtypes, year of NAC, and axillary surgery. Consequently, 388 patients were matched successfully (**Table 3**).

**Table 3:** Balanced statistics of patients receiving neoadjuvant chemotherapy in different surgical groups after propensity score matching.

Variables	Post-matching	
	Mastectomy group N = 194 (%)	Breast conservation group N = 194 (%)
**Age at diagnosis (years)**		
<55	162 (83.5)	165 (85.1)
≥55	32 (16.5)	29 (14.9)
**Years of NAC**		
2010-2011	13 (6.7)	12 (6.2)
2012-2013	21 (10.8)	19 (9.8)
2014-2015	53 (27.3)	48 (24.7)
2016-2017	48 (24.8)	50 (25.8)
2018-2019	59 (30.4)	65 (33.5)
**Menopausal status**		
Pre-menopausal	137 (70.6)	137 (70.6)
Post-menopausal	57 (29.4)	57 (29.4)
**ypT stage**		
T0/Tis	98 (50.5)	97 (50.0)
T1	80 (41.2)	79 (40.7)
T2	16 (8.2)	18 (9.3)
**ypN stage**		
N0	130 (67.0)	130 (67.0)
N1	45 (23.2)	45 (23.2)
N2	16 (8.2)	16 (8.2)
N3	3 (1.6)	3 (1.6)
**Molecular subtypes**		
Luminal A-like	16 (8.2)	16 (8.2)
Luminal B-like	93 (47.9)	95 (49.0)
HER2-positive	41 (21.1)	38 (19.6)
Triple-negative	44 (22.7)	45 (23.2)
**Axillary surgery**		
Sentinel lymph node biopsy	48 (24.7)	48 (24.7)
Axillary lymph node dissection	146 (75.3)	146 (75.3)

CBCS, conventional breast-conserving surgery; OBCS, oncoplastic breast-conserving surgery; ypT stage, post neoadjuvant therapy pathological tumor stage; ypN stage, post neoadjuvant therapy pathological node stage; pCR, pathological complete response; HER2, human epidermal growth factor receptor 2.

**Figure 4:**
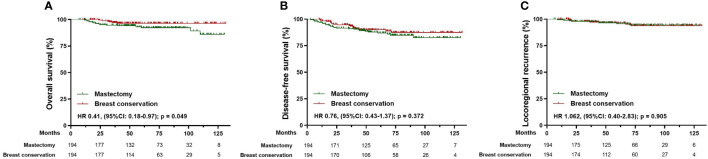
Survival analysis in the matched cohort of patients receiving mastectomy (n=194) and breast conservation (n=194). **(A)** Kaplan-Meier curves for overall survival analysis in patients of different surgical groups. **(B)** Kaplan-Meier curves for disease-free survival analysis in patients of different surgical groups. **(C)** Kaplan-Meier curves for locoregional recurrence analysis in patients of different surgical groups. HR, Hazard ratio; 95% CI, 95% confidence interval.

In the matched cohorts, the median follow-up time was 63.1 months for the mastectomy group and 55.0 months for the breast conservation group. Kaplan-Meier analysis showed that patients receiving breast conservation had better OS (HR 0.41, [95% CI 0.18-0.97]; p=0.049) compared with those who received mastectomy after NAC (**Figure 4A**). The 5-year OS for patients in the mastectomy group was 92.3% (95% CI 89.7%-93.8%) versus 96.4% (95% CI 94.5%-97.3%) in the breast conservation group. No statistical differences between the mastectomy and breast conservation group in 5-year DFS (86.9% versus 91.4%; HR 0.76, [95%CI: 0.43-1.37]; p=0.372) and LRR (95.6% versus 96.4%; HR 1.062, [95%CI: 0.40-2.83]; p=0.905) were observed (**Figures 4B, C**).

### Survival analysis between patients with and without application of oncoplastic techniques in surgery following NAC

We further compared the survival outcomes of patients with and without the application of oncoplastic techniques in the surgical process. Given the evolvement of surgical management, and substantial differences in the baseline of patients and tumor biologic characteristics between patients receiving CM-alone and M+IBR, or CBCS and OBCS, well-balanced groups were generated to reduce the selection bias for survival analysis.

**Figure 5:**
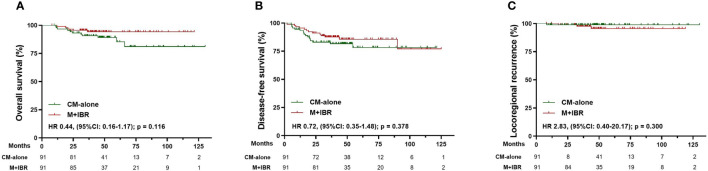
Survival analysis in the matched cohort of patients receiving conventional mastectomy alone (n=91) and mastectomy plus immediate breast reconstruction (n=91). **(A)** Kaplan-Meier curves for overall survival analysis in patients of different surgical groups. **(B)** Kaplan-Meier curves for disease-free survival analysis in patients of different surgical groups. **(C)** Kaplan-Meier curves for locoregional recurrence analysis in patients of different surgical groups. CM-alone, conventional mastectomy alone; M+IBR, mastectomy plus immediate breast reconstruction; HR, Hazard ratio; 95% CI, 95% confidence interval.

**Figure 6:**
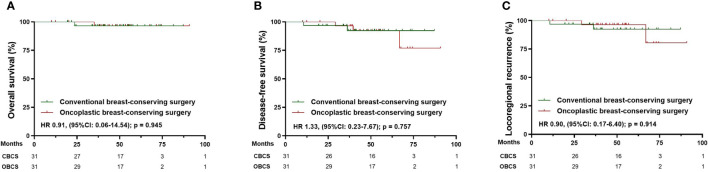
Survival analysis in the matched cohort of patients receiving conventional breast-conserving surgery (n=31) and oncoplastic breast-conserving surgery (n=31). **(A)** Kaplan-Meier curves for overall survival analysis in patients of different surgical groups. **(B)** Kaplan-Meier curves for disease-free survival analysis in patients of different surgical groups. **(C)** Kaplan-Meier curves for locoregional recurrence analysis in patients of different surgical groups. CBCS, conventional breast-conserving surgery; OBCS, oncoplastic breast-conserving surgery; HR, Hazard ratio; 95% CI, 95% confidence interval.

After matching, 91 patients receiving CM-alone and 91 patients receiving M+IBR were matched successfully (**Table 4**). The median follow-up was 48.1 months for the patients underwent CM-alone and 45.1 months for the patients underwent M+IBR. Data indicated no significant differences between the CM-alone and M+IBR groups in 5-year OS (85.3% versus 94.2%; HR 0.44, [95%CI: 0.16-1.17]; p=0.116), DFS (78.3% versus 85.6%; HR 0.72, [95%CI: 0.35-1.48]; p=0.378), and LRR (98.9% versus 95.5%; HR 2.83, [95%CI: 0.40-20.17]; p=0.300) (**Figure 5**). Regarding the breast conservation group, due to the relatively recent application of oncoplastic breast-conserving surgery in the NAC setting, matched patients were limited between CBCS (n=31) and OBCS (n=31) groups (**Table 4**). The median follow-up for patients who underwent CBCS and OBCS was 50.1 months and 50.1 months respectively. Moreover, no significant differences were identified in OS (96.4% versus 96.4%; HR 0.91, [95%CI: 0.06-14.54]; p=0.945), DFS (92.4% versus 92.2%; HR 1.33, [95%CI: 0.23-7.67]; p=0.757), LRR (92.4% versus 96.4%; HR 0.90, [95%CI: 0.17-6.40]; p = 0.914) between patients in those two groups (**Figure 6**).

**Table 4:** Matched patients receiving different surgical intervention in mastectomy group and breast conservation group after propensity score matching.

Variables	Mastectomy group (Matched)		Breast conservation group (Matched)	
	CM-alone N = 91 (%)	M+IBR N = 91(%)	CBCS N = 31 (%)	OBCS N = 31 (%)
**Age at diagnosis (years)**				
<55	84 (92.3)	83 (91.2)	27 (87.1)	28 (90.3)
≥55	7 (7.7)	8 (8.8)	4 (12.9)	3 (9.7)
**Years of NAC**				
2010-2011	3 (3.3)	4 (4.4)	–	–
2012-2013	7 (7.7)	6 (6.6)	–	–
2014-2015	14 (15.4)	14 (15.4)	4 (12.9)	5 (16.1)
2016-2017	12 (13.2)	12 (13.2)	16 (51.6)	15 (48.4)
2018-2019	55 (60.4)	55 (60.4)	11 (35.5)	11 (35.5)
**Menopausal status**				
Pre-menopausal	75 (82.4)	75 (82.4)	23 (74.2)	24 (77.4)
Post-menopausal	16 (17.6)	16 (17.6)	8 (25.8)	7 (22.6)
**ypT stage**				
T0/Tis	29 (31.9)	30 (33.0)	20 (64.5)	21 (67.7)
T1	38 (41.8)	38 (41.8)	9 (29.0)	8 (25.8)
T2	22 (24.2)	21 (23.1)	2 (6.5)	2 (6.5)
T3	2 (2.2)	2 (2.2)	–	–
**ypN stage**				
N0	45 (49.5)	48 (52.7)	24 (77.4)	24 (77.4)
N1	22 (24.2)	19 (20.9)	4 (12.9)	5 (16.1)
N2	17 (18.7)	17 (18.7)	2 (6.5)	1 (3.2)
N3	7 (7.7)	7 (7.7)	1 (3.2)	1 (3.2)
**Molecular subtypes**				
Luminal A-like	10 (11.0)	10 (11.0)	1 (3.2)	1 (3.2)
Luminal B-like	55 (60.4)	56 (61.5)	14 (45.2)	13 (41.9)
HER2-positive	12 (13.2)	12 (13.2)	7 (22.6)	8 (25.8)
Triple-negative	14 (15.4)	13 (14.3)	9 (29.0)	9 (29.0)
**Axillary surgery**				
SLNB	8 (8.8)	9 (9.9)	11 (35.5)	10 (32.3)
ALND	83 (91.2)	82 (90.1)	20 (64.5)	21 (67.7)

CM-alone, conventional mastectomy alone; M+IBR, mastectomy plus immediate breast reconstruction; CBCS, conventional breast-conserving surgery; OBCS, oncoplastic breast-conserving surgery; NAC, neoadjuvant chemotherapy; ypT stage, post neoadjuvant therapy pathological tumor stage; ypN stage, post neoadjuvant therapy pathological node stage; SLNB, sentinel lymph node biopsy; ALND, axillary lymph node dissection.

## Discussion

In the current study, patients who received breast conservation accounted for a small proportion of surgical management in the NAC setting and were identified with distinct features of demographic and clinicopathological variables compared with patients who underwent mastectomy. Moreover, we demonstrated comparable survival outcomes between patients receiving mastectomy and breast conservation, and further supported the application of oncoplastic techniques in the surgical intervention following NAC.

Neoadjuvant chemotherapy is increasingly used with extended indications, which brought consequential advantages and implications for de-escalation of local therapeutic decisions. Breast conservation is considered a reasonable option for selected patients receiving NAC in several guidelines (19, 20). Nevertheless, our results revealed an excessive inclination to mastectomy in the current clinical practice in China, which was accordant with another study conducted in northwest China (21). Moreover, the rate of breast-conserving surgery following NAC in the current study was much lower than research in Europe and the United States (7, 22). According to a recent retrospective study from Memorial Sloan-Kettering Cancer Center, the rate for patients successfully conducted with BCS after NAC was 44.96% (23). Likewise, Golshan et al. reported that 47.5% of patients with stage II-III triple-negative breast cancer underwent BCS after NAC in a prospective study, with a BCS conversion rate of 53.2% from ineligible to eligible owing to administration of NAC. Golshan et al. suggested that Asian and European patients showed significantly lower BCS rates, compared with patients in North America (24). However, our data revealed a steadily increasing tendency toward breast conservation, suggesting that our current surgical patterns may experience a similar shift from total mastectomy to breast conservation as earlier in those developed countries (10, 25).

Furthermore, with the comprehensive application of oncoplastic techniques in breast surgery, we revealed a considerable growing proportion of oncoplastic surgery in the NAC setting, including M+IBR and OBCS surgeries. As a combination of gland removal with breast skin preservation and immediate breast reconstruction, M+IBR was often called conservative mastectomy (26). Studies showed that M+IBR often correlated with improved patient satisfaction and aesthetic outcomes, which made it gain popularity in the selected patients (16, 27). We also reported a marked trend toward higher proportions of patients receiving M+IBR in the NAC setting. Regarding breast conservation, compared with CBCS, the addition of the oncoplastic technique in surgery allows a larger resection volume with rearrangement of resident breast tissue to achieve better cosmetic outcomes for the breast. Consequently, OBCS was increasingly utilized in patients eligible for breast conservation to further ensure complete tumor removal and correct breast deformities (28–30), and a similar trend was observed in patients undergoing NAC in our study. All these above indicated a rising need for improved cosmetic outcomes in patients receiving NAC. However, there were limited studies of matched groups on the efficacy and oncologic safety of oncoplastic surgery compared to conventional surgery in the NAC setting (31–33).

As for axilla management, ALND was more prevalent in the mastectomy groups, which implies an overall aggressive surgical paradigm in the NAC setting. Whereas, the proportion of SLNB increased appreciably among the patients who opted for breast conservation. In patients initially treated with surgery, SLNB offered good effectiveness in pathologic nodal staging and cancer control, along with a significant decline in arm morbidity, which led to a shift of axillary surgery from ALND to SLNB (34–36). Moreover, studies indicated that SLNB following NAC could achieve the acceptable false-negative rate (FNR) by sophisticated identification procedures, such as increasing the number of removed SLNs and applying of combined detection technique (37, 38). A growing number of studies further suggested that SLNB showed comparable oncologic safety in patients with clinically positive nodes in the NAC setting (39–41). Based on these clinical trials, SLNB was feasible and applicable after NAC for evaluation of residual disease in the axilla and local control. Consequently, considering the improvement of SLNB on arm function and quality of life, SLNB could be a reliable diagnostic alternative option for ALND for selected patients after NAC.

To determine the reasons that may contribute to the current surgical patterns, we compared characteristics between surgical groups. Consistent with recent research, we found that age and menopausal status could significantly influence surgical management after NAC (21). One possible explanation might be that elderly patients were often at weakened functional status with increasing comorbidities, resulting in extensive tension and anxiety about the disease and less concern about cosmetic appearance. Therefore, older patients were more likely to receive mastectomy to minimize the risk of recurrence and avoid the radiotherapy following BCS (42–44). Moreover, our data suggested that tumor biological features at presentation and after NAC were also variant among surgical options, including tumor size, disease stage, and molecular subtypes. Consistently, a growing number of evidence indicated that clinical tumor size was a significant predictor of pCR rate, which interferes with the preference of surgical options in patients administrated with NAC (23, 45–47). In addition, previous studies also demonstrated that patients diagnosed with triple-negative or HER2-positive breast cancer were less likely to be offered mastectomy (48). Petruolo O et al. and Golshan M et al. reported that HER2-positive and triple-negative were positively associated with successful downstaging of disease after NAC, which facilitated breast conservation (23, 49, 50).

Even though the mastectomy group had a higher proportion of older, post-menopausal patients with advanced tumor and disease stages before and after NAC, there was still a substantial number of patients who were eligible for breast conservation. In the current study, the pCR rate of patients with luminal A-like tumors was 5.2%, 20.4% for luminal B-like tumors, 51.7% for HER2-positive tumors, and 35.6% with triple-negative tumors, which were similar to the pCR rate in the ACOSOG Z1071 (Alliance) study and a German pooled analysis (22, 51). However, we found that more than 80% of patients who achieved pCR opted for mastectomy following NAC. Therefore, our findings suggested that disease-specific features were not the primary reason for the low rate of breast conservation. One of the possible explanations might be the lack of accurate and executable methods for the assessment of tumor regression in standard breast cancer management pathways (52). Additionally, according to the earlier studies, conservative attitude toward breast conservation in patients may also be an important factor that may lead to inclination toward mastectomy in the current surgical pattern, which implied insufficiency in evidence-based patient counseling (53).

To figure out whether the inclination toward mastectomy is necessary for patients treated with NAC, survival analysis was performed in matched cohorts of patients who underwent mastectomy or breast conservation. The survival outcomes of patients receiving NAC in the present study are in keeping with those in previous western studies (7, 54–56). Recently, Simons, J. M. et al. reported DFS of 90.9% for BCS and 82.9% for mastectomy, while OS of 95.3% for BCS and 85.9% for mastectomy (57). Moreover, our data showed a survival benefit on OS in patients who received breast conservation, while no significant difference was observed in DFS and LRR between the two groups, which were corroborated with the data of earlier studies with matched control groups (14, 56). Therefore, breast conservation should be considered as an alternative to mastectomy owing to better survival outcomes and equivalent locoregional control in the NAC setting.

In the present study, we further revealed comparable outcomes in well-matched cohorts of patients who underwent CM-alone and M+IBR. Previous studies also demonstrated the feasibility of M+IBR in terms of OS, DFS, and local recurrence. Zhen-Yu Wu, et al. compared oncologic outcomes between patients who underwent IBR with NSM/SSM and CM following NAC by a retrospective, propensity score-matched case-control study, which indicated comparable 5-year OS, DFS, LRR, and distant metastasis-free survival (32). And these findings were further confirmed in subgroups analysis of breast cancer patients with young age or locally advanced breast cancer (58, 59). In addition, our results further supported the oncologic safety of OBCS in the NAC setting. Compared with patients who underwent CBCS groups, we observed no differences in OS and DFS, along with comparable locoregional control in patients who received OBCS. A matched-cohort analysis even reported that OBCS could be a valuable option for tumors that regressed sub-optimally after NAC without compromising oncologic safety (33). Regarding locoregional control, a meta-analysis of two NSABP neoadjuvant trials indicated that LRR was not related to surgical options (54). Moreover, Chen et al. demonstrated an equivalent rate of negative margins in patients with large tumor size between OBCS and CBCS groups (60). Besides, wider resection margins in locally advanced tumors were also reported in OBCS (61). Hence, expanding the indication of oncoplastic techniques in conventional surgical intervention following NAC were feasible for patient administrated with NAC.

There were several limitations in the current study that should be taken into consideration while deducing the results. The most important limitation was caused by the retrospective study design. Since patients were retrospectively involved and divided into different surgical groups, the selection bias of surgical management was inevitable. Accordingly, we reported substantial heterogeneity in clinical features between patients undergoing mastectomy and breast conservation following NAC. In addition, considerate progression in surgical intervention and NAC regimens for breast cancer patients have occurred in recent decades, which lead to substantial differences in terms of the available treatment for patients. As a single cancer center in Shanghai, we had more than 2,000 breast cancer patients diagnosed and hospitalized every year since 2010, and started to perform oncoplastic surgery in 1999 (62). Therefore, oncoplastic breast surgery was performed by highly qualified breast surgeons who had undergone a long period of professional training in our institute. However, the application of oncoplastic techniques in surgical management after NAC started relatively late. Consequently, M+IBR and OBCS were new surgical options for patients with breast cancer receiving NAC, and they have lately gained popularity. Under this situation, it is critical to ensure that patients involved in the survival analysis were offered comparable therapies. Altogether, to minimize the inherent bias on the patient baseline, tumor biology, and treatment characteristics, nested case-control studies were required, and PSM was performed to balance such variations and generate matched cohorts for survival analysis. Also, the regression rate of tumor after NAC and original cup size were not included in the present study, which were also presumed to be potential impact factors for the surgical options. Moreover, the patient cohort of the current study was from a single institution. Therefore, although this cohort represents a real-world practice, multi-institutional investigations were required to further validate our results.

Altogether, although a growing proportion of patients opted for breast preservation after NAC, a considerate inclination toward mastectomy was observed in the current clinical practice in China. Moreover, we revealed a substantial evolvement in the application of the oncoplastic technique in surgical management following NAC. Our results strengthen the evidence that breast conservation was a feasible and safe alternative for mastectomy, which provided evidence to support surgical de-escalation in the NAC setting. Therefore, patient-centered clinical trials to guide treatment decisions, qualified pre-surgical assessment of tumor regression, thorough consultation of challenges and benefits of surgical de-escalation, along with a collaborative decision-making process between surgeons and patients, were necessary to improve the rate of breast conservation and quality of life for breast cancer patients receiving NAC.

## Data availability statement

The raw data supporting the conclusions of this article will be made available by the authors, without undue reservation.

## Author contributions

Conception and design: YS, BY, SH, XH, GL, ZS, and JW; administrative support: JC, BY, GL, ZS, and JW; provision of study materials or patients: YS, WC, and BY; collection and assembly of data: YS, WC, and XZ; data analysis and interpretation: YS and JW; manuscript writing: All authors. All authors contributed to the article and approved the submitted version.

## Funding

This study was supported in part by grants from the National Key R&D Program of China (2017YFC1311004).

## Conflict of interest

The authors declare that the research was conducted in the absence of any commercial or financial relationships that could be construed as a potential conflict of interest.

## Publisher’s note

All claims expressed in this article are solely those of the authors and do not necessarily represent those of their affiliated organizations, or those of the publisher, the editors and the reviewers. Any product that may be evaluated in this article, or claim that may be made by its manufacturer, is not guaranteed or endorsed by the publisher.
